# Both Splice Variants of Zebrafish Tmem11 Localize to the Outer Membrane of Mitochondria

**DOI:** 10.17912/micropub.biology.001162

**Published:** 2024-07-31

**Authors:** Saron S Tadesse, Melanie Schille, Paula Collado Cordon, Susan Walsh

**Affiliations:** 1 Life Sciences, Soka University of America; 2 Life Sciences, Mayo Clinic Comprehensive Cancer Center (Minnesota), Rochester, Minnesota, United States; 3 Department of Biology, Trinity College of Arts and Sciences, Duke University

## Abstract

In mammalian and *Drosophila* systems, Transmembrane protein 11 (TMEM11) regulates mitochondrial morphology, mitophagy, and mitochondrial function. Here, we begin to expand these studies to the zebrafish model system. We identified two splice variants of *tmem11* , which are both expressed during early development. In addition, we determined that both zebrafish Tmem11 proteins localize to the mitochondria using fluorescent tags and expression in cell culture. Consistent with recent data, biochemical fractionation indicates that Tmem11 is embedded in the outer membrane of mitochondria. Overall, these studies will provide new insights into the complex protein network that mediates mitochondrial physiology in the zebrafish.

**Figure 1. Zebrafish Tmem11 is an outer membrane mitochondrial protein:**
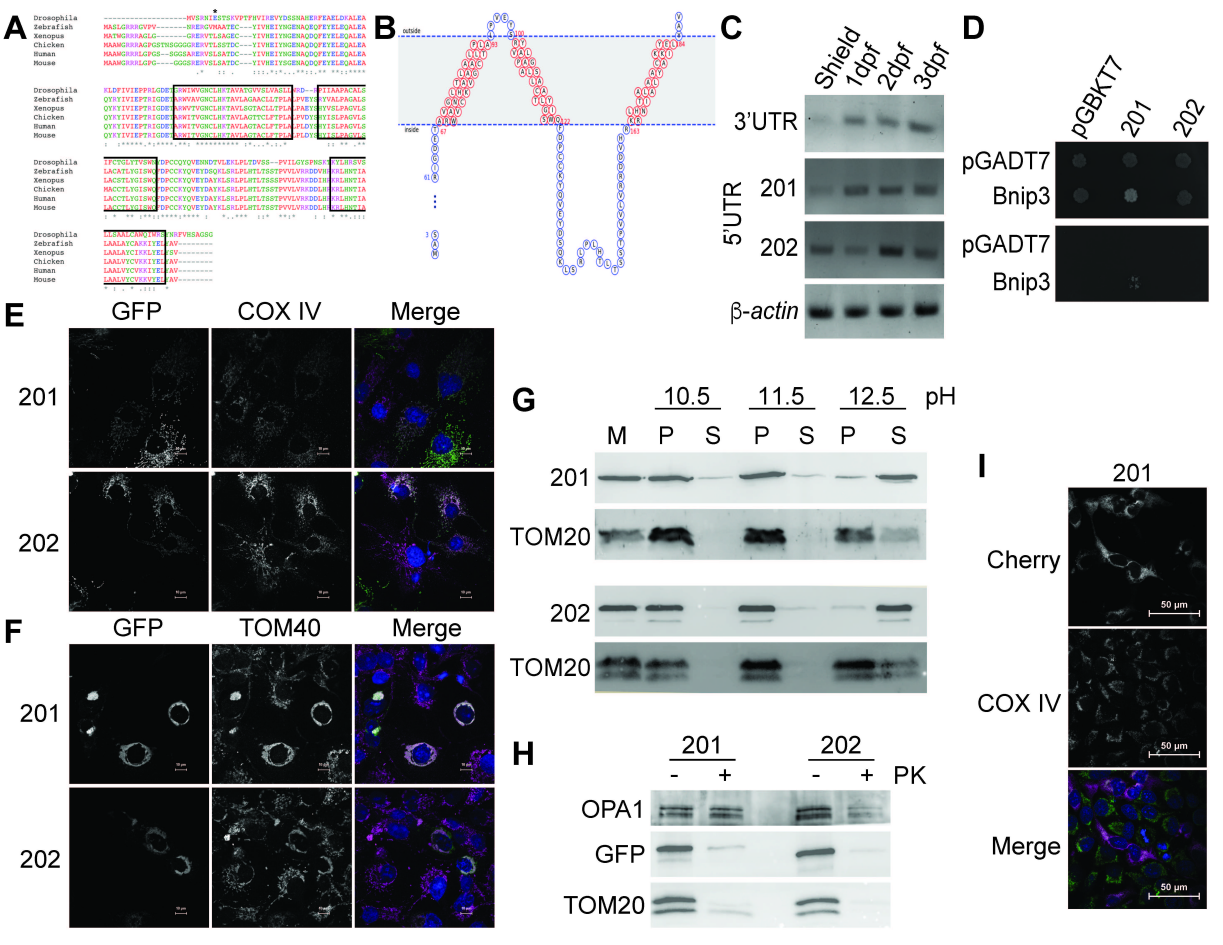
**A)** Uniprot protein sequences from human (P17152), mouse (Q8BK08), chicken (Q5ZLD4), zebrafish (201, Q6NWH5), fruit fly (Q8IQ56), and *Xenopus laevis* (A2BD92) were aligned in MUSCLE using ClustalW (Madeira et al. 2022). Boxed regions are predicted transmembrane domains in the zebrafish sequence. The asterisk marks the starting methionine of the splice variant of *tmem11* (202) that is missing the first 18 amino acids. **B)** MemBrain predicted topology indicating three transmembrane domains is shown (Feng et al. 2020). **C** ) RT-PCR using primers to the shared 3’ UTR of both *tmem11-201* and *202* or distinct 5’UTR primers demonstrate that both splice forms are expressed throughout early development in the zebrafish. *β-actin* is included as a control. **D)** Zebrafish Bnip3 interacts with Tmem11-201, but not Tmem-202, in a yeast two-hybrid. The top panel is selective only for the presence of plasmid (SD-Leu-Trp), but the bottom panel is selective for interactions (SD-Leu-Trp-His-Ade). Negative controls of vector without bait (pGBKT7) or vector without prey (pGADT7) are included. **E)** Both N-terminally EGFP-tagged Tmem11 proteins localize to the mitochondria of COS7 cells, as shown by counterstaining with the mitochondrial marker COX IV. **F)** High expression of the GFP-tagged Tmem11 proteins in COS7 cells cause highly fused mitochondria, as shown by counterstaining with the mitochondrial marker TOM40. **G)** Both EGFP-tagged Tmem11 proteins are embedded in the mitochondrial membrane (P) and not easily solubilized (S) based on carbonate extraction (pH 10.5, 11.5, or 12.5) of purified mitochondria (M) from transfected cells. An antibody against GFP was used to detect both proteins. TOM20 served as a control membrane protein. **H)** Both EGFP-tagged Tmem11 proteins localize to the outer mitochondrial membrane, as shown by treatment of purified mitochondria from transfected cells with Proteinase K (PK). OPA1 is a mitochondrial inner membrane protein which is protected from Proteinase K, whereas TOM20, on the outer membrane, is degraded. **I)** A C-terminally Cherry-tagged Tmem11-201 protein does not localize to the mitochondria of HeLa cells, as shown by counterstaining with the mitochondrial marker COX IV.

## Description

The transmembrane (TMEM) protein family vaguely describes any proteins that contain at least one putative transmembrane domain which fully spans a biological membrane (Beasley et al. 2021, Marx et al. 2020) . This protein family has been implicated in cellular functions as diverse as cell division, metastasis, calcium regulation, and mitochondrial dysfunction. Transmembrane protein 11 (Tmem11) is a known regulator of mitochondrial morphology (Macchi et al. 2013; Rival et al. 2011) . The knockdown of *TMEM11* in HeLa cells shifts mitochondrial morphology from tubular structures to spherical, enlarged structures (Rival et al. 2011) . A similar phenotype was observed in flies when the *TMEM11* ortholog, Pantagruelian Mitochondrion I ( *PMI* ), was mutated, resulting in excessive fission and a reduction in the number of the mitochondria (Rival et al. 2011) . TMEM11 interacts with some proteins of the mitochondrial contact site and cristae junction organizing system (MICOS), consistent with its role in mitochondrial morphology and cristae organization (Guarani et al., 2015) . Additionally, *PMI* mutant flies had impaired respiratory chain function, reduced life span, and impaired neuronal activity (Macchi et al. 2013) . In mice, knocking out *TMEM11* increased cardiomyocyte proliferation and cardiac function, while overexpression of *TMEM11* inhibited cardiomyocyte proliferation and cardiac regeneration (Chen et al. 2023) . Although previously characterized as a mitochondrial inner membrane protein (Rival et al. 2011) , human TMEM11 has recently been found to localize to the outer membrane of the mitochondria using super-resolution microscopy (Gok et al. 2023) . There, it interacts with the mitophagy mediators BNIP3 and BNIP3L to regulate spatial specificity of hypoxia-induced mitophagy (Gok et al. 2023, Rual et al., 2005) . Recently, zebrafish is emerging as a model for mitophagy studies (Feng et al. 2011; Pant and Nazarko 2021; Wrighton et al. 2021) . Given the connection of TMEM11 to mitophagy, we sought to determine whether the zebrafish Tmem11 proteins also localized to the mitochondria as a starting point for understanding this process.

Through Ensembl, we identified the zebrafish protein ortholog (ENSDARG00000070866) and its two splice variants with a shared 3’ end: *201* , the longer form, and *202* , which utilizes a start site 18 amino acids downstream of the initiator methionine of the *201* form ( Figure 1A ). Similar to the human protein (Gok et al. 2023) , both zebrafish Tmem11 proteins are predicted to be transmembrane proteins which cross the membrane three times ( Figure 1B ; Feng et al. 2020). Not surprisingly for a predicted mitochondrial protein, high throughput *in situ* hybridization data suggested that zebrafish Tmem11 protein is ubiquitously expressed (Thisse and Thisse 2004) , and non-quantitative RT-PCR using primers to the shared 3’UTR or to the different 5’UTRs of *201* and *202* demonstrate that both these proteins are likely expressed throughout early development ( Figure 1C ). In addition, using yeast two-hybrid, we explored whether the zebrafish Tmem11 proteins bind to zebrafish Bnip3, an interaction found previously in humans (Gok et al. 2023) . Only Tmem11-201, but not Tmem11-202 (or vector-only negative controls), grew on selective media when zebrafish Bnip3 was also expressed ( Figure 1D ), indicating an interaction between these two proteins. As this is a heterologous system, it is unclear whether *in vivo* the additional 18 amino acids at the N-terminus of Tmem11-201 are truly responsible for this interaction. Based on data with the human proteins demonstrating that the transmembrane domains, and not the N-terminus, are necessary and sufficient for the interaction (Gok et al. 2023) and since we do not have a known interactor for this particular construct, it seems like a lack of interaction with the Tmem11-202 protein in this system might be an artifact.

To determine the subcellular localization of zebrafish Tmem11, we tagged both splice variants of Tmem11 on the N-terminus with EGFP and transfected them into COS7 cells for microscopy studies. The cells were stained with the mitochondrial markers COX IV and TOM40, and proteins from both transcripts of Tmem11 appeared to localize to the mitochondria ( Figure 1E and 1F). Notably, at higher expression of both proteins (as determined by the intensity of the signal), the mitochondria were clumped together ( Figure 1F ). This phenotype is consistent with overexpression of human TMEM11 in HeLa cells and indicates a defect with mitochondrial fission or fusion (Rival et al. 2011) . In contrast, a C-terminally tagged Tmem11-Cherry did not co-localize with the mitochondrial marker ( Figure 1I ), and we suspect that this tag may interfere with appropriate integration of the last transmembrane domain into the mitochondrial membrane ( Figure 1B ). To complement our imaging data and further determine which submitochondrial compartment zebrafish Tmem11 localizes to, we conducted biochemical fractionation experiments using HeLa cells transfected with the EGFP-tagged Tmem11 proteins. Mitochondrial fractions were treated with carbonate to extract proteins from the membranes; soluble proteins or peripherally associated membrane proteins are associated with the supernatant at lower pHs than integral membrane proteins which remain in the pellet even at a high pH (Claypool et al., 2006, Fujiki et al., 1982) . We observed that both variants of zebrafish EGFP-Tmem11 were associated with the membrane fraction ( Figure 1G ), consistent with the predictions ( Figure 1B ). In order to determine whether Tmem11 was on the outer membrane or the inner membrane of mitochondria, mitochondrial fractions were treated with Proteinase K to degrade outer membrane proteins, and westerns for GFP (Tmem11), TOM20 (outer membrane marker), or OPA1 (inner membrane marker) were used to detect the remaining proteins. Notably, Tmem11-201 ran at a slightly higher molecular weight than Tmem11-202, as predicted, since 201 includes an additional 18 amino acids. Both EGFP-Tmem11 proteins and TOM20 were significantly degraded when treated with Proteinase K, but OPA1 was not, indicating that these proteins are on the outer membrane of mitochondria, like the mammalian ortholog ( Figure 1H ; Gok et al. 2023). Both OPA1 and TOM20 appear as two bands on the western, as previously reported when using these particular antibodies (Koma et al, 2021, Chang et al, 2023) .

Although our experiments represent overexpression in a heterologous system, which can cause mitochondrial morphology defects, the clarity of our results, in addition to the predicted transmembrane domains and lack of a canonical nuclear localization sequence (Nguyen Ba et al., 2009) , indicate that zebrafish Tmem11 is localized to the outer membrane of the mitochondria, which is consistent with its human ortholog (Gok et al. 2023) . Interestingly, mouse TMEM11 is additionally localized in the nucleus and cytoplasm in cardiomyocytes (Chen et al. 2023) . This discrepancy could be explained due to differences in organismal systems and/or tissue-specificity. In mouse cardiomyocytes, Chen et al (2023) propose that TMEM11 does not affect mitochondrial membrane potential, apoptosis, or mitophagy, but does regulate the transcription factor ATF5, which moves from the mitochondria to the nucleus after impaired mitochondrial import. These data are distinct from the mitophagy regulation through interactions with BNIP3 and BNIP3L reported by Gok et al. (2023) for human TMEM11. Whether zebrafish Tmem11 regulates hypoxia-induced mitophagy in skeletal muscle might be assessed *in vivo* through CRISPR knockouts of one or both *tmem11* splice variants in the context of the mitophagy biosensor zebrafish (Wrighton et al. 2021) . Additional future research on Tmem11 in zebrafish will involve delineating if the splice variants have distinct functions or expression and the physiological role of Tmem11 in regulating mitophagy, mitochondrial morphology, and developmental biology.

## Methods

***RT-PCR***

Total RNA from zebrafish AB embryos at various developmental stages (shield, 1 day post fertilization (dpf), 2 dpf, or 3 dpf) was isolated using TRIzol (Invitrogen). Luna Universal One-Step RT-qPCR (New England Biolabs) was used with 0.5µg total RNA from each developmental stage according to manufacturer’s directions. Primer pairs amplified the two distinct 5’UTRs and the shared 3’UTR. β-actin was used as a positive control (Walsh et al, 2017) . PCR products were resolved on a 1.5% agarose gel with 100bp ladder (New England Biolabs).

***Cloning***

gBlocks (Integrated DNA Technologies) containing the zebrafish *tmem11-201* gene sequence flanked by *EcoRI* and *BamHI* restriction sites or the zebrafish *bnip3* gene sequence flanked by *XhoI* and *BamHI* restriction sites was cloned into pmCherry2-N1 (gift from Michael Davidson (Addgene plasmid # 54517; http://n2t.net/addgene:54517 ; RRID:Addgene_54517)). To clone *tmem11-201* into pEGFP-C1 (Clontech), *tmem11-201* was amplified with zTMEM11.SalI.F and zTMEM.BamHI.R using Q5 DNA polymerase (New England Biolabs) at annealing temperature of 65°C. pEGFP-C1 was digested with *XhoI* and *BamHI* . Ligation of compatible cohesive ends from *SalI* and *XhoI* restriction enzymes ablated both sites. To clone the zebrafish *tmem11-202* , the zebrafish *tmem11-201* pEGFP-C1 plasmid was amplified using LongAmp Taq DNA Polymerase (New England Biolabs) using the primers zTMEM11-202.splice.F and pEGFPC1.splice.R using an annealing temperature of 58°C, phosphorylated with T4 Polynucleotide Kinase (New England Biolabs), ligated with T4 DNA ligase (New England Biolabs), treated with DpnI (New England Biolabs) to degrade template DNA, and transformed into NEB-𝛼 cells. After sequencing (Eurofins Genomics), the gene was out of frame with the EGFP, and a QuikChange Lightning Kit (Agilent) with zTMEM202.SDM.F and zTMEM202.SDM.R was used to restore the frame. Plasmids were propagated in NEB5-𝛼 cells (New England Biolabs) and purified using the PureLink HiPure Plasmid Midiprep Kit (Invitrogen K210004) before transfection into HeLa cells (ATCC CCL-2) or COS7 cells (ATCC CRL-1651).

For yeast two hybrid, *bnip3* was cloned into pGADT7 (prey, Matchmaker Gold, Takara Bio) from *bnip3* pmCherry2-N1 using the primers zBNIP3.Y2H.EcoRI.F and zBNIP3.Y2H.BamHI.R. *tmem11-201* and *202* were cloned into pGBKT7 (bait, Matchmaker Gold, Takara Bio) from *tmem11-201* pEGFP-C1 using the primers zTMEM11.Y2H.EcoRI.F for *201* or zTMEM11-202.splice.F for *202* and zTMEM11.Y2H.BamHI.R for both variants.

***Yeast Two-Hybrid***

A high efficiency transformation (Gietz and Schiestl, 2007) was used to transform bait and prey plasmids into Y187 or Y2H Gold yeast, respectively (Matchmaker Gold, Takara Bio). Yeast were inoculated in YPD (1% yeast extract, 2% peptone, 2% dextrose) and incubated overnight on a rotary shaker at 200 rpm at 30ºC. Cells were then added to 50 mL pre-warmed 2X YPAD (2% yeast extract, 4% peptone, 4% dextrose, 0.008% adenine). When the cell titer was 2 x 10 ^7^ cells/mL, the cells were harvested at 3000 *xg* for 5 minutes, washed and resuspended in sterile water. The suspension was centrifuged, and the supernatant was discarded. Sterile water was added to a final volume of 1 mL to resuspend the cells. For each transformation, 100 µL yeast were centrifuged and resuspended in 360 µL transformation mix (33.33% PEG 3500, 0.1 M lithium acetate, boiled salmon sperm carrier DNA, and 2-5 µg plasmid DNA). Transformations were incubated at 42ºC for 40 minutes. The transformation mix was removed by centrifugation, and 1 mL sterile water was added to each tube. Yeast were plated onto SD-Leu or SD-Trp (0.05% ammonium sulfate, 0.17% yeast nitrogen base, 2% dextrose, dropout mix, 2% agar) and incubated for 3-4 days at 30ºC. When the colonies were grown, the yeast were mated by combining them in 0.5mL 0.5X YPAD for 24 hours at 30ºC. The mated yeast were plated onto SD-Leu-Trp and incubated for 3-4 days at 30ºC. Subsequent diploid colonies were grown in SD-Leu-Trp liquid media at 30ºC, diluted to an OD _600_ of 0.0005, and 5 µL were spotted onto SD-Leu-Trp-His-Ade+X-𝛼-gal or SD-Leu-Trp for incubation at 30ºC for 3-4 days until colonies grew, and blue color developed.

***Immunofluorescence***

Cells were split onto No. 1.5 sterile glass coverslips (Electron Microscopy Sciences) in a 6-well plate and grown in DMEM + 10% FBS media at 37℃, 5% CO _2_ . Once the cells were greater than 60% confluent, they were transfected using Lipofectamine ^TM^ 3000 based on the manufacturer’s instructions (Invitrogen). The cells were incubated at 37°C overnight and fixed with 100% ice-cold methanol. Fixed cells were washed three times with 1X phosphate buffered saline (PBS) for 5 minutes and blocked in 5% normal goat serum/0.3% Triton X/PBS for 1hr at RT. The primary antibodies COX IV or TOM40 in 0.3% TritonX/PBS were added to the cells, and they were incubated at 4℃ overnight. After three PBS washes, the cells were incubated in the secondary antibody in 0.3% TritonX/PBS for 1 hour at RT. Cells were stained with DAPI diluted 1:10000 in PBS for 5 minutes and washed twice with 1X PBS for 5 minutes. They were imaged using a Nikon C2 confocal microscope at 600X magnification.

***Fractionation of Mitochondria***

Four 10 cm plates of HeLa cells were transfected with either *tmem11* /pEGFP-C1. Twenty-four hours post transfection, cells were washed once with 1X PBS, trypsinized, and collected in PBS by spinning at 3000 *xg* for 5 minutes. The cell pellet was resuspended in 1 mL STE (250 mM sucrose, 5 mM Tris-HCl pH 7.5, 1 mM EGTA) with protease inhibitors (Cepham Life Sciences) and 1 mM DTT and incubated on ice for 30 minutes. The resuspended cells were then homogenized with a Teflon dounce (size 20) 50 times on ice. Broken cells were centrifuged at 680 *xg* for 10 minutes at 4℃. The supernatant containing the mitochondria was moved to a new tube, and the mitochondria were pelleted by centrifugation at 14000 *xg* for 10 minutes at 4℃. Supernatant containing the cytosol was moved to a new tube and centrifuged at full speed (21000 *xg* ) for 10 minutes at 4℃ to remove any remaining particulates. Mitochondrial pellets were gently resuspended in 1mL STE with protease inhibitors and DTT and centrifuged at 11000 *xg* for 10 minutes at 4℃. The supernatant was discarded, and the mitochondrial pellet was resuspended in STE using a microfuge pestle.

To determine if TMEM11 was embedded in a membrane, isolated mitochondria were resuspended in STE and centrifuged at 8000 *xg* for 10 minutes at 4℃. Pellets were resuspended in 0.1M carbonate solution at a basic pH (pH 10.5, 11.5, or 12.5). After 30 minutes on ice, mitochondria were centrifuged at 21000 *xg* for 10 minutes at 4℃, and the supernatants were removed. Trichloroacetic acid (TCA) was added to the supernatants to a final concentration of 20% and incubated on ice for 1 hour. The pellets were resuspended in Thorner buffer (40 mM Tris-HCl pH 8, 5% SDS, 8 M urea, 100 µM EDTA, 715 mM β-mercaptoethanol). After 1 hour, precipitated soluble proteins were spun for 10 minutes 4℃ at 21000 *xg* , washed once with acetone, and resuspended in Thorner buffer.

To determine whether Tmem11 was on the outer membrane, mitochondria were resuspended in STE or STE with 100 µg/mL Proteinase K. After 30 minutes on ice, 0.2M PMSF was added to a final concentration of 25 mM, and the mitochondria were harvested by centrifugation at 21000 *xg* for 10 minutes at 4℃. Supernatant was discarded, and the pellets were resuspended in 40 µL SDS-PAGE Laemmli sample buffer.

Samples were heated at 95℃ for 2 minutes and resolved by SDS-PAGE with Tricolor Prestained Protein Marker (Bioland Scientific LLC) before transfer to PVDF. Primary antibodies were GFP, TOM20, COX IV, or OPA1. The membrane was imaged using the ChemiDoc ^TM^ MP Imaging System (Biorad) and Clarity ^TM^ Western ECL Substrate (Biorad).

## Reagents

Primers and gBlocks (Integrated DNA Technologies)

**Table:** 

Name	Sequence	Purpose
zTMEM11.SalI.F	5’-TAA GTCGAC TAATGGCGTCGCTGGGAAG	Cloning *tmem11-201*
zTMEM.BamHI.R	5’-TAA GGATCC ATACAGCGTACAGTTCAT	Cloning *tmem11-201*
zTMEM11-202.splice.F	5’-GTGATGGCGGCCACG	Cloning *tmem11-202*
pEGFPC1.splice.R	5’-AGATCTGAGTCCGGACTTGTAC	Cloning *tmem11-202*
zTMEM202.SDM.F	5’-CAAGTCCGGACTCAGTCTTGTGATGGCGGC	Cloning *tmem11-202*
zTMEM202.SDM.R	5’-GCCGCCATCACAGACTGAGTCCGGACTTG	Cloning *tmem11-202*
*tmem11-201* gBlock	5’-CATGAT GAATTC TAATGGCGTCGCTGGGAAGGAGGCGCGGTGTCCCAGTCAACAGGGAGAGGGGAGTGATGGCGGCCACGGAGTGTTACATCGTCCACGAGATCTACAACGGCGAGAATGCACAGGAGCAGTTCGAGTACGAGCTGGAGCAGGCTCTGGAGGCGCAATACCGCTACATCGTGATCGAGCCCACGCGGATCGGGGACGAGACGGCGCGATGGGTAGCAGTCGGAAATTGTCTGCATAAGACAGCAGTGCTTGCAGGAGCAGCTTGCCTCCTGACGCCGCTCGCCCTCCCCGTCGAATACTCCCGTTACGTGGCGCTGCCGGCTGGCGCTCTGAGCTTGGCCTGTGCCACGCTGTACGGCATATCCTGGCAGTTCGACCCCTGCTGCAAGTACCAGGTGGAGTACGACAGTCAGAAGCTCTCGCGGCTGCCCCTGCACACACTCACCTCCTCCACGCCGGTGGTTCTTGTCCGACGGGACGACGTGCACAGAAAGAGACTGCACAACACGATAGCGTTGGCGGCCCTCGCGTACTGTGCCAAGAAGATCTATGAACTGTACGCTGTAAT GGATCC ATGCTG-3’	Cloning *tmem11-201*
β-actin.F	5′-ATCAGGGTGTCATGGTTGGT	Control RT-PCR
β-actin.R	5′-CACGCAGCTCGTTGTAGAAG	Control RT-PCR
zTMEM11.3'UTR.RT.F	5’-TCCTTTCCCCTCCCTCGC	RT PCR for 3’UTR
zTMEM11.3'UTR.RT.R	5’-ATGTGACGCACCAAAGGC	RT PCR for 3’UTR
zTMEM11.5UTR.R	5’-CGATGTAGCGGTATTGCGCC	RT PCR for 5’UTR
zTMEM11.201.5UTR.F	5’-GCGTCAAGTCTAGTCCGTTG	RT PCR for 5’UTR of *tmem11-201*
zTMEM11.202.5UTR.F	5’-GCAGTTGTTATAACACGGTTTTCCC	RT PCR for 5’UTR of *tmem11-202*
zTMEM11.Y2H.EcoRI.F	5’-ATCA GAATTC ATGGCGTCGCTGG	Cloning into Y2H vector pGBKT7
zTMEM11.Y2H.BamHI.R	5’-ATC GGATCC TCATACAGCGTACAGTTCATAG	Cloning into Y2H vector pGBKT7
zBNIP3.Y2H.EcoRI.F	5’-ATCA GAATTC ATGTCGATTGAAAAGCACAGCG	Cloning into Y2H vector pGADT7
zBNIP3.Y2H.BamHI.R	5’-ATT GGATCC CTACCCGAGACCCACAG	Cloning into Y2H vector pGADT7
zBNIP3.Xho.Bam gBlock	5’-​​ATCT CTCGAG ATGTCGATTGAAAAGCACAGCGTGTCTGAAGAAAACCTTCAGGGTTCTTGGGTGGAGCTGCATTTTAATAATGGGGGAGGCAGCACTTCTAAAGCAGCCACAGATGAACAGTCAGCTAGCACGGCCCCGAGCGGAGACCTGGAGAAGATGCTTCTGGATGCTCAGCACGAGTCGGGTCGGAGCAGCTCCAGAGGAAGCCTACCATGTGACAGTCCTCCAAGATCCCAGACTCCTTTGCACTTGTGCAGGGGCTCCGAGGTCCACAGCTCTGGGGAAAAAAACAGCTCACAGTCAGAGGAAGACTATTTGGAGAGGAGAAAAGAGGTGGAGATCCTGATGAAGAAAAATGCTGATTGGATCTGGGACTGGTCGAGTCGCCCAGAAAACCTGCCACCCAAGGAGTTTCTGCTGCGGCACCCGAAGCGCTCCAGCACACTCAGCATGAGGAACACCAGTGTGATGAAGAAAGGAGGAATCTTCTCTGCTGAGTTCCTCAAAGTCTTCCTGCCCTCTCTGGTCCTCTCACACATCCTCGCTGTGGGTCTCGGGAT GGATCC AGC	Cloning zBNIP3 into pmCherry2-N1

Antibodies

**Table:** 

Antigen	Animal and Clonality	Dilution	Source	Purpose
COX IV	Rabbit polyclonal	1:500 (IF) 1:1000 (western)	Cell Signaling Technology 3E11, Catalog #4850	IF, western
TOM40	Rabbit polyclonal	1:1000	Generous gift of CM Koehler, UCLA	IF
GFP	Mouse monoclonal	1:1000	Santa Cruz Technology B2, sc-9996	western
TOM20	Rabbit polyclonal	1:500	Santa Cruz Biotechnology FL-145 Catalog #sc-11415	western
OPA1	Rabbit polyclonal	1:1000	Abclonal, Catalog #A9833	western
anti-mouse rhodamine	Goat polyclonal	1:1000	Jackson Immunochemicals	IF
anti-rabbit rhodamine	Goat polyclonal	1:1000	Jackson Immunochemicals	IF
anti-rabbit FITC	Goat polyclonal	1:1000	Jackson Immunochemicals	IF
anti-mouse HRP	Goat polyclonal	1:10000	Jackson Immunochemicals	western
anti-rabbit HRP	Goat polyclonal	1:10000	Jackson Immunochemicals	western

Plasmids

**Table:** 

Gene	Plasmid backbone	Description
*tmem11-201*	pEGFPC1	Zebrafish *tmem11, transcript 1* cloned in frame with EGFP at the N-terminus. Available from authors.
*tmem11-202*	pEGFPC1	Zebrafish *tmem11, transcript 2* cloned in frame with EGFP at the N-terminus. Available from authors.
*tmem11-201*	pmCherry2-N1. Available from Addgene plasmid # 54517	Zebrafish *tmem11, transcript 1* cloned in frame with Cherry2 at the C-terminus. Available from authors.
*tmem11-201*	pGBKT7	Zebrafish *tmem11, transcript 1* cloned in frame for Y2H. Available from authors.
*tmem11-202*	pGBKT7	Zebrafish *tmem11, transcript 2* cloned in frame for Y2H. Available from authors.
*bnip3*	pGADT7	Zebrafish *bnip3* cloned in frame for Y2H. Available from authors.
*bnip3*	pmCherry2-N1. Available from Addgene plasmid # 54517	Zebrafish *bnip3* cloned in frame with Cherry2 at the C-terminus. Available from authors.
